# Angiosperms and the Linnean shortfall: three new species from three lineages of Melastomataceae at one spot at the Atlantic Forest

**DOI:** 10.7717/peerj.1824

**Published:** 2016-03-21

**Authors:** Renato Goldenberg, Fabián A. Michelangeli, Lidyanne Y.S. Aona, André M. Amorim

**Affiliations:** 1 Departamento de Botânica, Universidade Federal do Paraná, Curitiba, Paraná, Brazil; 2 Institute of Systematic Botany, The New York Botanical Garden, Bronx, NY, United States of America; 3 Centro de Ciências Agrárias, Ambientais e Biológicas, Universidade Federal do Recôncavo da Bahia, Cruz das Almas, Bahia, Brazil; 4 Departamento de Ciências Biológicas, Universidade Estadual de Santa Cruz, Ilhéus, Bahia, Brazil; 5 Herbarium CEPEC, Comissão Executiva do Plano da Lavoura Cacaueira, Ilhéus, Bahia, Brazil

**Keywords:** Angiosperms, Brazil, Huberia, *Meriania*, Bahia, *Physeterostemon*

## Abstract

Three new species of Angiosperms have been found in four short collection trips to the same protected reserve—“Estação Ecológica Estadual de Wenceslau Guimarães”—and neighboring areas in the Atlantic Forest in the south of the Brazilian state of Bahia. These new species belong to three genera from three distinct lineages in the family Melastomataceae: *Huberia*, *Meriania* and *Physeterostemon*. The description of these species represent a good example of a Linnean shortfall, i.e., the absence of basic knowledge about the biodiversity in the area, as well as in tropical forests as a whole. The description of these probably endemic species per se is a signal that this area deserves more attention regarding research and policies, but its consequences go farther: this area has a relevant role as a phylogenetic (both genetic and morphological) stock, and thus is also valuable as a phylogenetic conservation priority.

## Introduction

Studies on biodiversity and evolutionary biology have been traditionally hindered by gaps in basic, natural history knowledge, no matter what kind of methods or organisms they deal with. These “shortfalls” can be linked to the lack of taxonomic work, i.e., species descriptions (“Linnean shortfall”; Brown & Lomolino, 1998; Whittaker et al., 2005) or absence of data regarding their distribution (“Wallacean shortfall”; Bini et al., 2006). Both lead to a third gap in our knowledge: the lack of information on phylogenetic relationships, and thus the evolutionary history of life on Earth (i.e., “Darwinian shortfall”, Diniz-Filho et al., 2013).

The forests of eastern Brazil (Atlantic Forest or “Mata Atlântica”) harbor an incredible amount of biodiversity, and are considered one of the biodiversity hotspots (Myers et al., 2000). With less than 10% of the original vegetation remaining, the Atlantic Forest is also a high conservation priority (Myers et al., 2000; Ribeiro et al., 2009). Our knowledge about biodiversity in this area is not static as inventories of angiosperms have shown. In 2009 the list of flowering plants from the Atlantic Forest contained ca. 13,700 species (Stehmann et al., 2009), while the most recent account (BFG, 2015) includes ca. 15,000 species. In both cases 49–49.5% of the species are reported as endemic. This 1,300 species increase within six years is mainly due to the participation of specialists for most plant families and the heavy use of new informatic tools (BFG, 2015). However, this increase also includes new species being described at a steady rate. In a 16 year span (1990–2006), 1,194 species of angiosperms were described for the Atlantic Forest (Sobral & Stehmann, 2009), which represents an increase of about 80 species per year. Taking the total 13,700 species surveyed in 2009, this means that almost 8.7% of these species were described in the previous 15 years.

This high species description rate of angiosperms has been more intense in the middle section of Atlantic Forest from southern Bahia trough Espírito Santo and North Eastern Rio de Janeiro. The forests in that range are very rich for both plants (Martini et al., 2007; Leitman et al., 2015), and animals (Carnaval & Moritz, 2008; Carnaval et al., 2009; Silva et al., 2012). Moreover, these areas have been historically poorly collected, and thus lesser known than the southern portions of Atlantic Forest in São Paulo, Paraná and Santa Catarina, and central and western Rio de Janeiro. Recent collection efforts, especially in medium to high altitudes have yielded several new species (for Melastomataceae only: Amorim, Goldenberg & Michelangeli, 2009; Goldenberg & Reginato, 2009; Baumgratz, Amorim & Jardim, 2011; Reginato, Baumgratz & Goldenberg, 2013; Amorim, Jardim & Goldenberg, 2014). This was also the case for our four short, 3–4-day collection trips to small area within the municipality of Wenceslau Guimarães, in southern Bahia. These trips provided enough material for the description of three new species of Melastomataceae, from three different genera: *Huberia* DC., *Meriania* Sw. and *Physeterostemon* R. Goldenb. & Amorim. These genera belong to distinct lineages, respectively the *Merianthera* clade (Goldenberg et al., 2012), tribe Merianieae (Goldenberg et al., 2015), and Miconieae/*Eriocnema* clade (Amorim, Goldenberg & Michelangeli, 2009). *Huberia* has 16 species, most of them from eastern Brazil except for four species found in the Andes in Peru and Ecuador (Baumgratz, 2004). *Meriania* has 95–110 species, ranging from southern Mexico and Caribbean to Bolivia and southeastern Brazil (Michelangeli, Carmenate-Reyes & Sosa, 2015). *Physeterostemon* has four species, all endemic to southern Bahia (Amorim, Jardim & Goldenberg, 2014).

The discovery of three distantly related species in the same family and in the same area underscores our lack of knowledge for several groups and areas of the tropics. It is important to emphasize that every one of the three species described here has characters that are unique within their respective lineages (see discussions below), thus the discovery of these new species goes beyond that of adding to species to groups and areas, but also greatly add to our knowledge of morphological diversity and evolution for each of the three genera. Moreover, some of the groups described in the last 10 years represent key lineages heretofore unknown. An example is *Physeterostemon* (Goldenberg & Amorim, 2006), first described from two species from southern Bahia, and that turned out to represent the sister group of the Miconieae (Amorim, Goldenberg & Michelangeli, 2009), an exclusively Neotropical group with ca. 2,000 species.

The description of the species here is as a good example of a Linnean shortfall, because three new species from a single family are found in the same place, disclosing the absence of basic knowledge regarding the biodiversity of the area. As for conservation purposes, the description of these probably endemic species *per se* is a signal that this area deserves more attention regarding research and policies, but its consequences go farther: this area has a relevant role as a phylogenetic (both genetic and morphological) stock (Vane-Wright, Humphries & Williams, 1991; Faith, 1992; Nee & May, 1997; Sechrest et al., 2002; Hidasi-Neto, Loyola & Cianciaruso, 2013), and thus is also valuable as a phylogenetic conservation priority.

## Study Area

**Figure 1:**
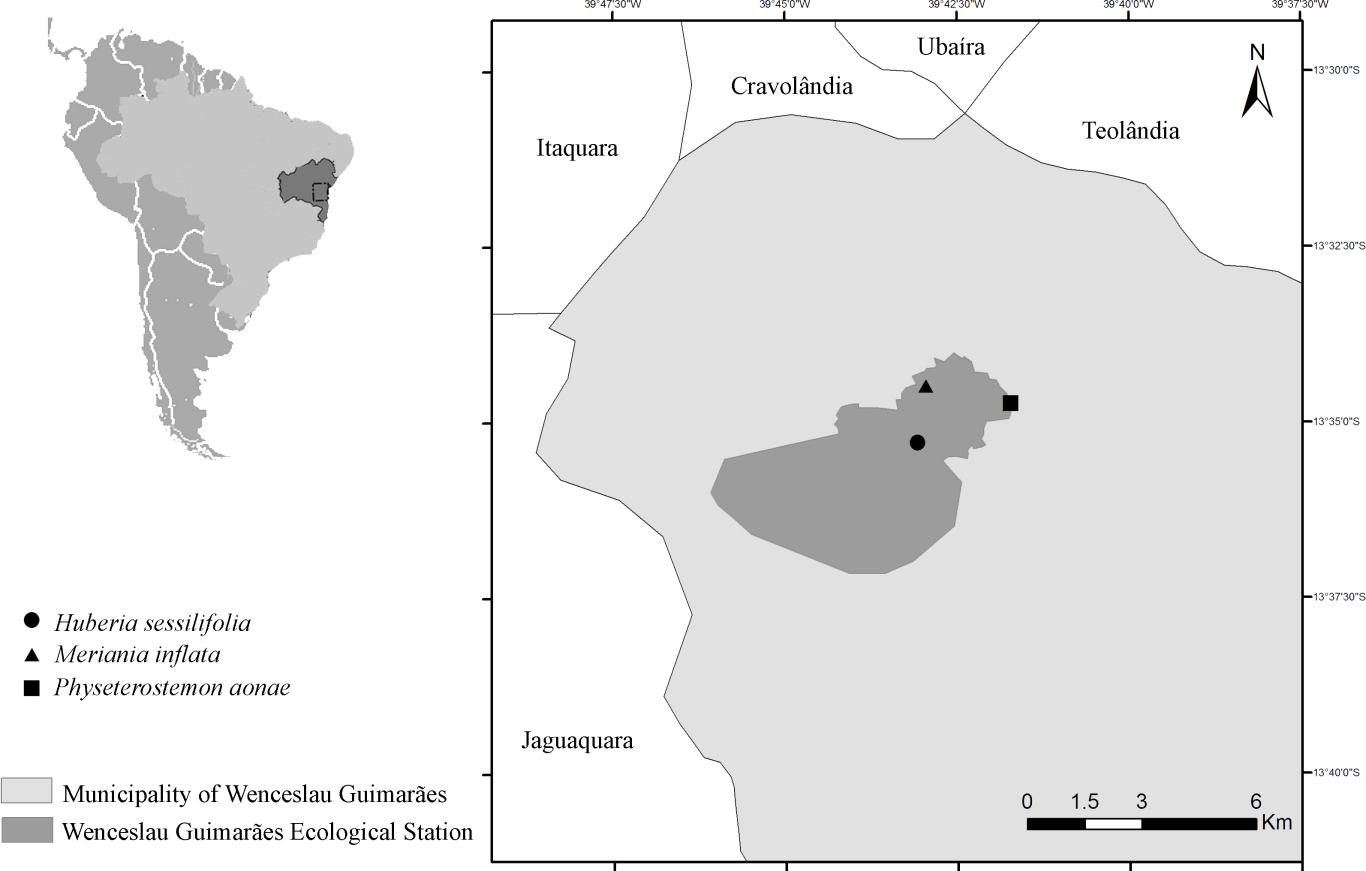
Map with the collection localities in the municipality of Wenceslau Guimarães, Bahia, Brazil.

The “Estação Ecológica Estadual de Wenceslau Guimarães” (EEEWG) was created in 1997 and enlarged in 2000, totaling 2,418 hectares (Bahia, 2010). The reserve is contained within a single municipality, Wenceslau Guimarães, in the southern portion of the state of Bahia, Brazil (Fig. 1). Its geomorphology includes deep and straight valleys 200–500 m wide, at 550 m elevation at the lowest point, and enclosed by mountains up to 1,000 m elevation, with slopes ranging mostly between 15° and 50°. The soils are mostly alic Oxisols, but there are also Lithic Psamments and Ultisols. The climate is tropical and humid, but some slopes facing west can be considered as sub-humid. The annual mean temperature ranges between 22° and 25.5 °C, and rainfall between 800 and 1,500 mm (Bahia, 2010).

The area surrounding the reserve has been almost entirely logged and cleared. It is nowadays covered with small rural properties, and the agricultural activities are based mostly on cocoa, fruticulture (mostly soursop, locally called “graviola,” and sold as frozen pulp) and subsistence crops. Some areas inside the reserve has been cleared and used for agricultural purposes for a long time up to the 1990s, but now there are no families living in it anymore. Apart from some areas covered with secondary vegetation, mostly on the bottom of the valleys and lower slopes, the reserve is covered with sub-montane tropical moist forest (“Floresta Ombrófila Densa Submontana,” according to the Brazilian official classification system; Veloso, Rangel-Filho & Lima, 1991). On slopes up to 50°, the canopy is 8–15 m high, and average trunk diameter 15–30 cm, with remnant trees up to 30 m tall and 1 m diameter; the understory is well developed and the trees usually heavy with epiphytes. The vegetation on slopes above 800 m is lower, the canopy at 3–6 m, and average trunk diameter 5–10 cm, with a dense moss and lichen cover. Steeper and more exposed slopes and some mountain tops are covered with sparse vegetation, with a mosaic of shrubs and herbs on lithic soils.

Botanical exploration in the area is recent. They began in the early 1990s, with A.M. Carvalho, F. França, E. Melo, S. Mayo, W.W. Thomas and S.C. Sant’Anna. Surprisingly there are only three new species of plants described with types from Wenceslau Guimarães, all of them bromeliads (Leme & Luther, 1998; Leme, 1999; Leme & Luther, 2003). There is also a new species of treefrog (Anura) described for the area (Caramaschi & Rodrigues, 2003).

## Material and Methods

Fertile specimens were collected and processed following the traditional procedures related to botanical specimens (Mori et al., 1989). The holotypes and paratypes were deposited either at CEPEC or UPCB (acronyms following Thiers, 2015), with duplicates sent to HURB, NY, RB and other herbaria, whenever available. Comparisons with other species in each genus were based in herbarium specimens and bibliography as well: Baumgratz (1997, 2004; *Huberia*); Chiavegatto (2009) and Chiavegatto & Baumgratz (2015; *Meriania*); Goldenberg & Amorim (2006), Amorim, Goldenberg & Michelangeli (2009), Amorim, Jardim & Goldenberg (2014; *Physeterostemon*). Our judgment on the species limits followed the morphological-phenetic species concept (Judd, 2007). Collection permits were issued by the “Instituto do Meio Ambiente e Recursos Hídricos” (INEMA/Bahia: 2014-008132/TEC/PESQ-0010 and 2015-010036/TEC/PESQ-0015) for the plants inside “Estação Ecológica Estadual de Wenceslau Guimarães.”

The electronic version of this article in Portable Document Format (PDF) will represent a published work according to the International Code of Nomenclature for algae, fungi, and plants (ICN), and hence the new names contained in the electronic version are effectively published under that Code from the electronic edition alone. In addition, new names contained in this work which have been issued with identifiers by IPNI will eventually be made available to the Global Names Index. The IPNI LSIDs can be resolved and the associated information viewed through any standard web browser by appending the LSID contained in this publication to the prefix “http://ipni.org/”. The online version of this work is archived and available from the following digital repositories: PeerJ, PubMed Central, and CLOCKSS.

**Figure 2:**
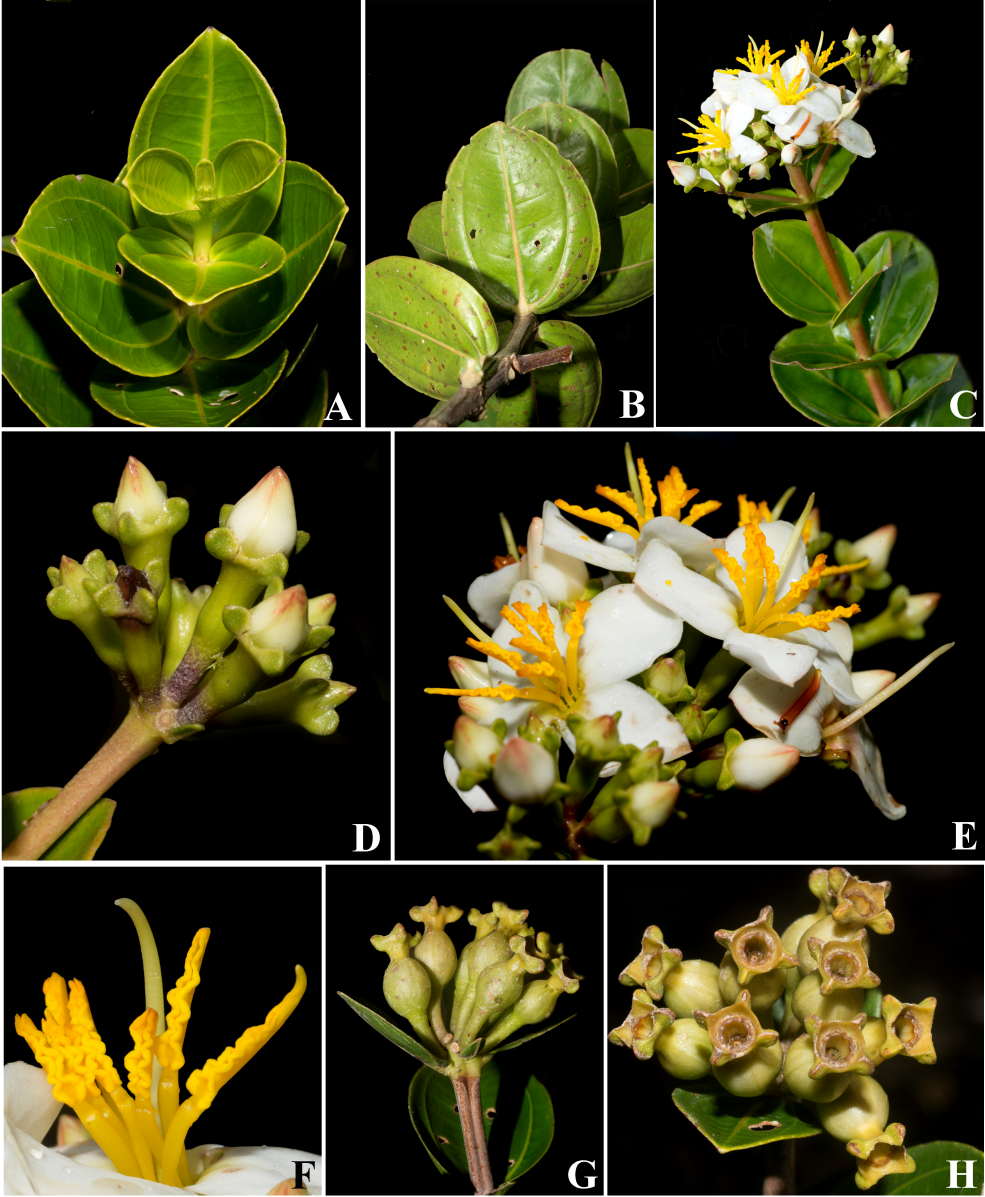
*Huberia sessilifolia*. (A) Vegetative branch, adaxial view. (B) Vegetative branch, abaxial view. (C) Flowering branch. (D) Inflorescence with flowers in bud. (E) Inflorescences with flowers at anthesis. (F) Stamens and style. (G) Infrutescence with capsules before dehiscence, side view. (H) Infrutescence with capsules before dehiscence, top view (all photos by Fabian Michelangeli).

**Figure 3:**
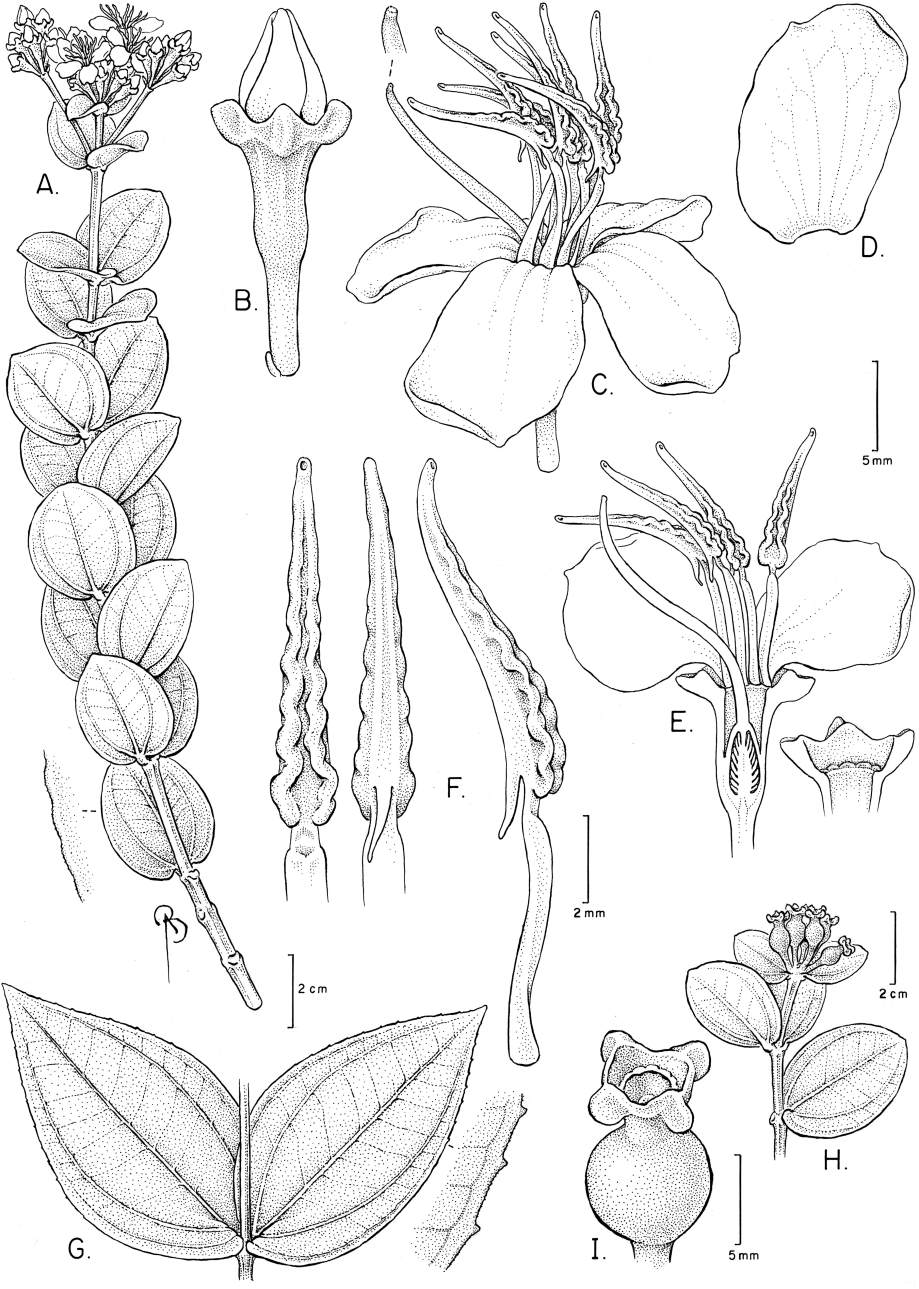
*Huberia sessilifolia*. (A) Flowering branch. (B) Flower bud. (C) Flower at anthesis. (D) Petal, adaxial surface. (E) Flower, longitudinal section, and detail of torus with petals and anthers removed. (F) Anthers in ventral, dorsal and side view. (G) Leaves of lower branches with detail of the leaf margin. (H) Infrutescence. (I) Fruit, before dehiscence. (A–F from Goldenberg et al. 2052, NY; G–I from Goldenberg et al. 1762, NY, and also from alcohol-preserved flowers and photos of live plants).

**Figure 4:**
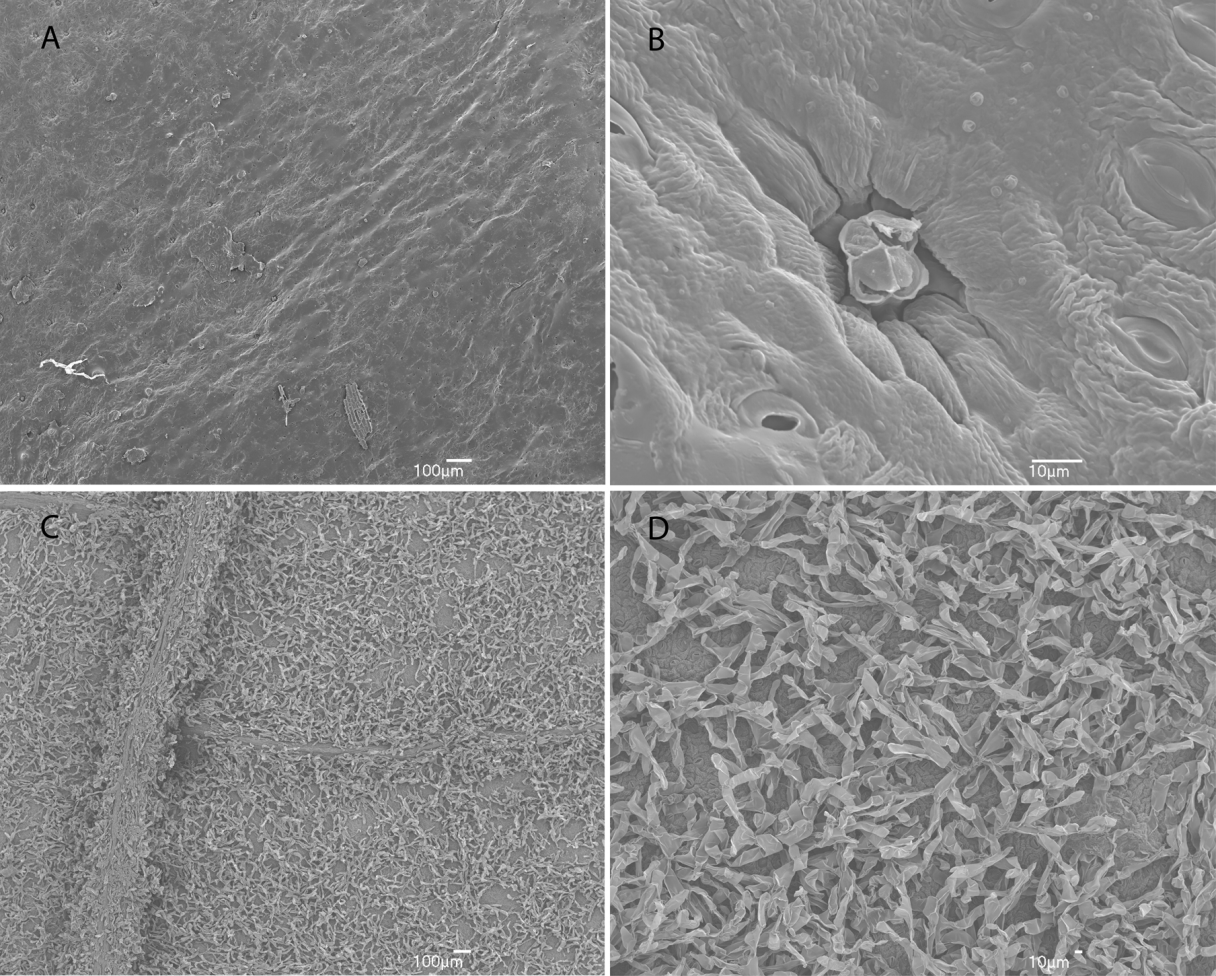
SEM’s of leaf surfaces of *Huberia sessilifolia* (A–B) and *Meriania inflata* (C–D).

## Results

***Huberia sessilifolia*** R. Goldenb. & Michelangeli, spec. nov.

(Figs. 2, 3 and 4A–4B)

**Type**. Brazil, Bahia: Mun. Wenceslau Guimarães, Estação Ecológica Estadual Wenceslau Guimarães, trilha para a Pedra da Torre, 13°35′17.1^′′^S, 39°43′4.3^′′^W, 750 m, 12 Oct 2014 (fl), *R. Goldenberg* & *F. A. Michelangeli 2052* (holotype: UPCB; isotypes: CEPEC, HURB, K, MBML, MO, NY, RB).

**Diagnosis**. A species of *Huberia* that differs from all other species in the genus due to the sessile leaves with subcordate to cordate bases, short (1–1.3 mm long) sepals, and the connective with a very short (1.3–1.7 mm long) dorsal tooth.

**Description**. Trees or shrubs 4–5 m high, with few erect branches. Young stems, leaves, inflorescences and hypanthia glutinose (both in fresh and dry specimens), covered with glandular trichomes with very short stalks, which are more distinctive on both leaf surfaces, where they are located on depressions and look more like dots than trichomes, otherwise glabrous. Young stems slightly quadrangular or slightly 4-sulcate, later becoming terete; interpetiolar lines absent. Leaves opposite, isomorphic, bigger on the proximal portions of the branches, then gradually smaller towards the apex; petioles absent when seen in adaxial view, but the leaves have a thick, rounded, 1–1.5 mm long protuberance at the base, in abaxial view, that may be interpreted as a reduced petiole; blades on fertile branches (2-)3–5 × (1-)1.7–3.7 cm, chartaceous, ovate to ovate-elliptic, base subcordate to cordate, apex broadly acute to obtuse or rounded, margin entire and narrowly hyaline; blades on sterile branches larger than on the fertile branches, up to 9 × 5 cm, apex obtuse to acute, margins denticulate; venation acrodromous, basal, lacking domatia, with one pair of secondaries, elevated on abaxial surface, and with an additional pair of faint submarginal veins, tertiary veins percurrent but barely perceptible, quaternaries and areoles not perceptible in both surfaces. Inflorescence usually a single terminal, erect, 7–13-flowered umbellate cyme, 2–2.7 cm long, with a pair of bracts 1.4–2.3 × 0.9–1.3, leafy, sessile, oval to lanceolate, apex rounded, on a peduncle 1.6–2.2 cm long, or the inflorescences with one central and two lateral axes (totaling 20–30 flowers), each one similar to the cyme described above, but the laterals on peduncles 2–3 cm long, lacking bracts; bracteoles 2 per cluster, 2.3–7 × 1–2 mm, sessile, oblong to lanceolate, apex rounded. Flowers 4-merous, on pedicels 5.5–7 mm long. Hypanthium green, 5.5–6.5 × 2–2.5 mm, terete to oblong-urceolate, surface smooth (not alate, nor costate). Calyx opening regularly; tube 0.6–0.9 mm tall; sepals 1–1.3 mm long, broadly deltoid; dorsal teeth 0.5–0.7 mm. long, longitudinally flattened, triangular to rounded (then more prominent and rounded in fruits). Petals white, 11–13 × 8–9.5 mm, asymmetrically obovate, the apex rounded but with a sub-apical tooth, papillose. Stamens 8, isomorphic, all bent to to one side of the flower at anthesis, yellow but turning orange–red in older flowers; filaments 6–7.5 mm long; connective not extended below the thecae but with a dorsal, straight, subulate tooth 1.3–1.7 mm long; thecae 7.7–8.5 mm long, sigmoid, corrugated, opening by a ventrally inclined pore. Ovary 4–4.5 mm long, ca. 1/2 superior, 4-locular; style sigmoid, white or pale-yellow, 13–14.5 mm long, tapering towards the apex, bent to the same side of the flower than the anthers; stigma punctiform. Young capsules (“ruptidio” sensu Baumgratz, 1985), yellowish-green, 10–12 mm long on pedicels 9–11 mm long, urceolate, surface smooth (not alate nor costate), with four longitudinal faint nerves plus four alternating shallow furrows. Mature fruits and seeds not seen.

**Distribution and conservation status.** *Huberia sessilifolia* is known from only one population along an exposed rocky ridge within the “Estação Ecológica Estadual Wenceslau Guimarães” in southern Bahia. Given the size of the reserve (24.2 km^2^) and potential habitat, we recommend that *H. sessilifolia* is given a status of “critically endangered” (IUCN, 2014).

**Paratypes**. Brazil, Bahia: Mun. Wenceslau Guimarães, Estação Ecológica Estadual Wenceslau Guimarães, 730 m, 21 Feb 2014 (fr), *R. Goldenberg et al.* 1762 (CEPEC, NY, UPCB).

**Etymology**. The epithet refers to the sessile leaves, which are the most distinctive feature of this species.

**Remarks**. All species of *Huberia* known to date have petiolate leaves, with bases ranging from cuneate and decurrent to obtuse to rounded (Baumgratz, 1997). No described species present the sessile leaves with subcordate to cordate bases found in *H. sessilifolia*. The character combination of short sepals and short connective appendages can be found in *H. glazioviana* Cogn. and *H. consimilis* Baumgratz. However in these species the connective appendage ranges from 1.5 to 4 mm long in the former, and 1.3 to 4.7 mm long in the latter (Baumgratz, 1997), thus usually longer than the 1.3–1.7 mm long ones found in *H. sessilifolia*. Apart from the petiolate leaves with acute to obtuse bases, *H. glazioviana* also differ from *H. sessilifolia* on the pubescent branches and leaves, while *H. consimilis* differs from *H. sessilifolia* on the longer (up to 24 mm long) and slenderer pedicels (Baumgratz, 1997).

**Figure 5:**
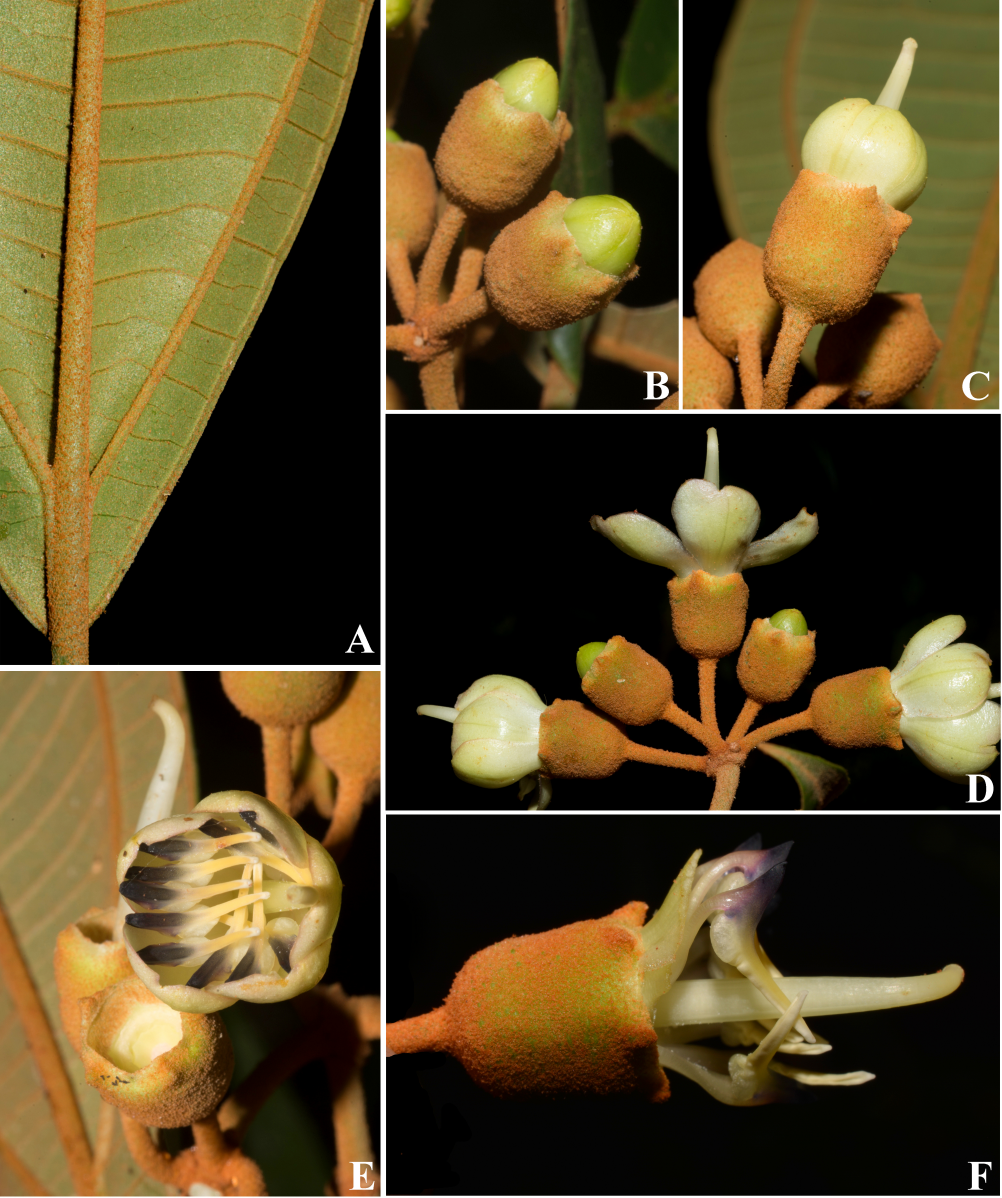
*Meriania inflata*. (A) Leaf blade, abaxial view. (B) Flower buds. (C) Flower bud in pre-anthesis. (D) Inflorescence with flowers in different development stages. (E) Flower, top view. (F) Flower, lateral view, petals removed (all photos by Fabian Michelangeli).

**Figure 6:**
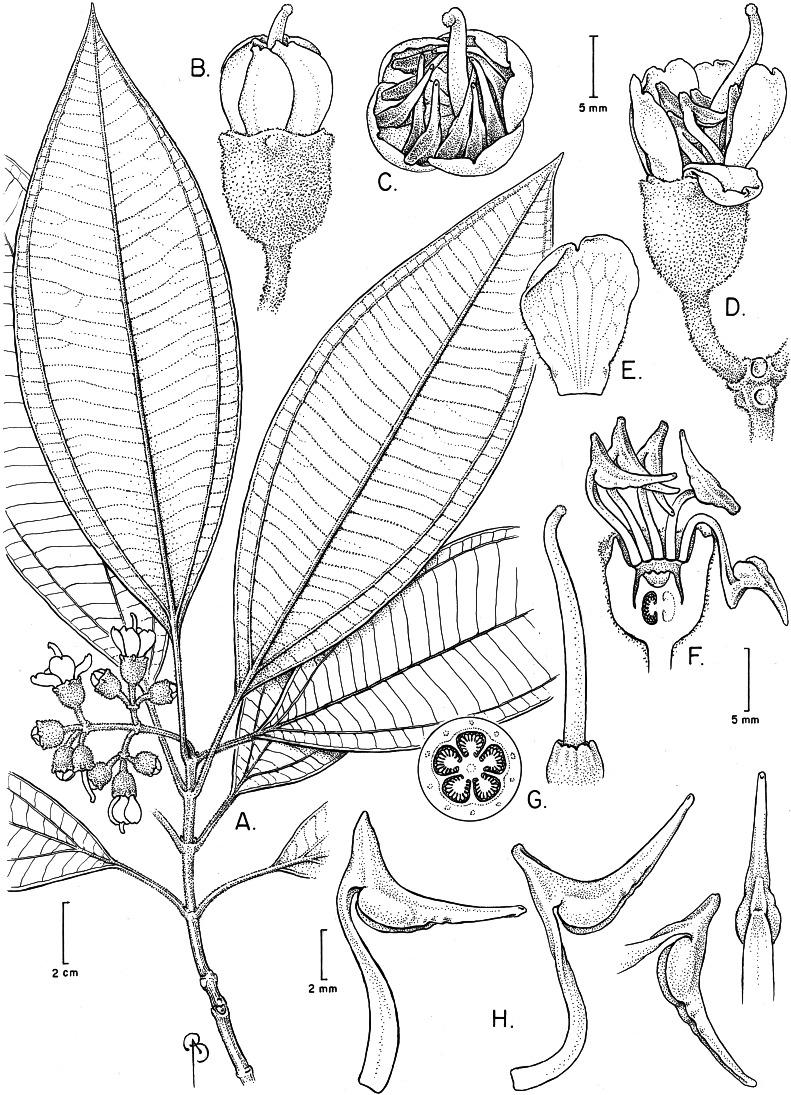
*Meriania inflata*. (A) Flowering branch. (B) Flower at anthesis, lateral view. (C) Flower at anthesis, top view. (D) Flower at anthesis with one petal removed, lateral view. (E) Petal adaxial surface. (F) Flower, longitudinal section with petals removed. (G) Ovary apex and style and ovary in cross section. (H) Stamens in side, dorsal and ventral view (all drawn from Goldenberg 2078, NY, alcohol-preserved flowers and photos of live plants).

***Meriania inflata*** Michelangeli & R. Goldenb., spec. nov.

(Figs. 4C–4D, 5 and 6)

**Type**. Brazil, Bahia: Mun. Wenceslau Guimarães, Estação Ecológica Estadual Wenceslau Guimarães, trilha do Alto da Água Vermelha, 13°34′28.3^′′^S, 39°42′58.8^′′^W, 675 m, 13 Oct 2014 (fl), *R. Goldenberg* & *F. A. Michelangeli 2078* (holotype: UPCB; isotypes: CEPEC, HUEFS, HURB, K, MBM, NY, RB, SP, US).

**Diagnosis**. A species of *Meriania* similar to *Meriania tetramera* Wurdack, but with flowers pentamerous and a regularly opening calyx with well defined sepals and dorsal teeth.

**Description**. Tree up to 16 m high. The young stems covered with dense amorphous rufous trichomes. Young stems quadrangular, pubescent, later becoming terete, glabrous, and the bark fisurate; interpetiolar lines present in younger nodes, not obvious later on. Leaves opposite, mostly isomorphic; petioles 1.5–4 (-5) cm long with dense trichomes as in the stem; blades 11.5–21 × 4–8.5 cm (the pair below the inflorescence often reduced to 4.5–6.5 × 1.8–2.5 cm), chartaceous, elliptic to narrowly elliptic, base acute to narrowly acute, apex acute to shortly acuminate (up to 12 mm), margin entire and slightly revolute; venation acrodromous, suprabasal, with one pair of secondaries diverging (3-) 8–15 mm above the base, often slightly asymmetric, and with an additional pair of faint submarginal veins, tertiary veins percurrent, spaced every 3–7 mm and faint quaternaries, the areoles 2–4 mm; abaxial surface obscured by the pubescence as in the stem, the veins elevated, with slightly longer trichomes than on the surface, the union of the primary and secondary veins forming pocket domatia; adaxial surface appearing glabrous, but with very sparse appressed simple trichomes <1 mm long, the veins impressed. Inflorescence a terminal few-flowered cyme, 5–8.5 cm long, deflexed or pendant, the flowers in dichasia or sub-umbellate clusters; penducles flattened. Flowers 5-merous, the pedicels 8–11 mm long. Hypanthium 4.5–5.5 × 5.5–6.8 mm, broadly campanulate, with dense trichomes as in the stem. Calyx opening regularly, with dense trichomes as in the stem; tube 1.8–2.5 mm tall; sepals 2.5–3.2 mm, broadly deltoid; dorsal teeth tuberculate, up to 1.8 mm long. Petals green in bud, pale green at anthesis, 9–12.5 × 6.7–8 mm, obovate, glabrous, the apex emarginated, the base truncate. Stamens 10, slightly dimorphic, all bent to to one side of the flower at anthesis (generally the lower side); filaments 7.5–8.5 mm long, white; connective pale yellow, not extended below the thecae but with a black dorsal acute tooth 2.8–3.3 mm long; thecae white, subulate, opening by a dorsally inclined pore, each theca with an inflated sac at the base, 2.3–3.3 mm long, white and translucent. Ovary 4/5 to fully superior, 5-locular; style slightly sigmoid, white, protogynous, 11–13.5 mm long, 0.9–1 mm diameter at the base, tapering towards the apex, bent to the opposite side of the flower than the anthers; stigma punctiform ca. 0.5 mm wide. Capsules and seeds unknown.

**Distribution and conservation status**. *Meriania inflata* is known from only one collection from forests within the Estação Ecológica Estadual Wenceslau Guimarães in southern Bahia. At the time of collecting the type specimen three individuals were observed. Given the size of the reserve (24.2 km^2^) and potential habitat, we recommend that *Meriania inflata* is given a status of “critically endangered” (IUCN, 2014).

**Etymology**. The epithet refers to the two inflated sacs at the base of the thecae. This character occurs in both *Meriania inflata* and *Meriania tetramera*, and makes these two species very distinctive among other species of *Meriania*.

**Remarks**. Among the Eastern Brazilian species of *Meriania*, the indument and anther morphology of *M. inflata* are very similar to those of *M. tetramera* (Chiavegatto, 2009; Chiavegatto & Baumgratz, 2008; Chiavegatto & Baumgratz, 2015). This anther morphology with paired sacs at the base of the thecae is not known in any other species in the genus apart from *M. inflata* and *M. tetramera*. This feature is undoubtedly associated with the pollination biology of these species, but nothing is known about the natural history of either species. While *Meriania tetramera* also has greenish petals and plinerved leaves, the two species can be readily distinguished because *Meriania tetramera* has 4-merous flowers and a calyx closed in bud that opens irregularly. The leaf and hypanthium indument of *M. inflata* is also similar to that of *M. calophylla* (Naudin) Triana. However, *M. calophylla* has narrower leaves that are basally-nerved, much larger flowers with purple to lavender petals at anthesis, and dimorphic stamens with dorsal ascending appendages. In the recent key to Brazilian *Meriania* (Chiavegatto & Baumgratz, 2015), *M. inflata* would not fit either of the options in the first couplet as the inflorescences are pendulous or deflexed (as in *M. glazioviana* Cogn. and *M*. *longipes* Triana) but the peduncle is less than 4 cm long (as in all other Brazilian species).

***Physeterostemon aonae*** Amorim, Michelangeli & R. Goldenb., spec. nov.

(Figs. 7 and 8)

**Figure 7:**
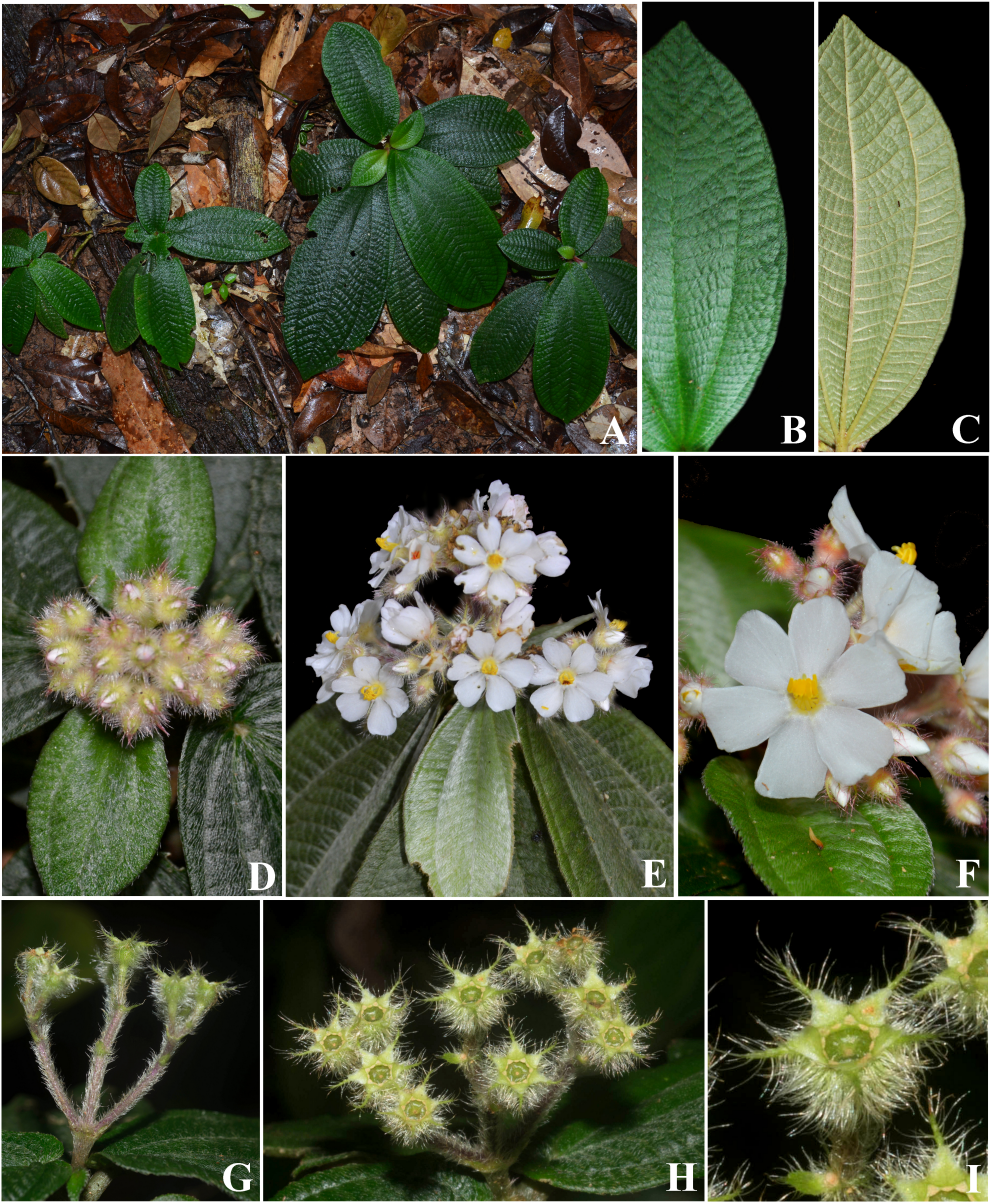
*Physeterostemon aonae*. (A) Habit of flowering plants. (B) Leaf adaxial surface. (C) Leaf, abaxial surface. (D) Inflorescence with flowers still in bud. (E) Inflorescence with flowers at anthesis. (F) Flower, frontal view. (G) Infrutescence, lateral view. (H) Infrutescence, top view. (I) Fruit. (A, D–F by André Amorim, B–C by Lucas Marinho, G–I by Fabian Michelangeli).

**Figure 8:**
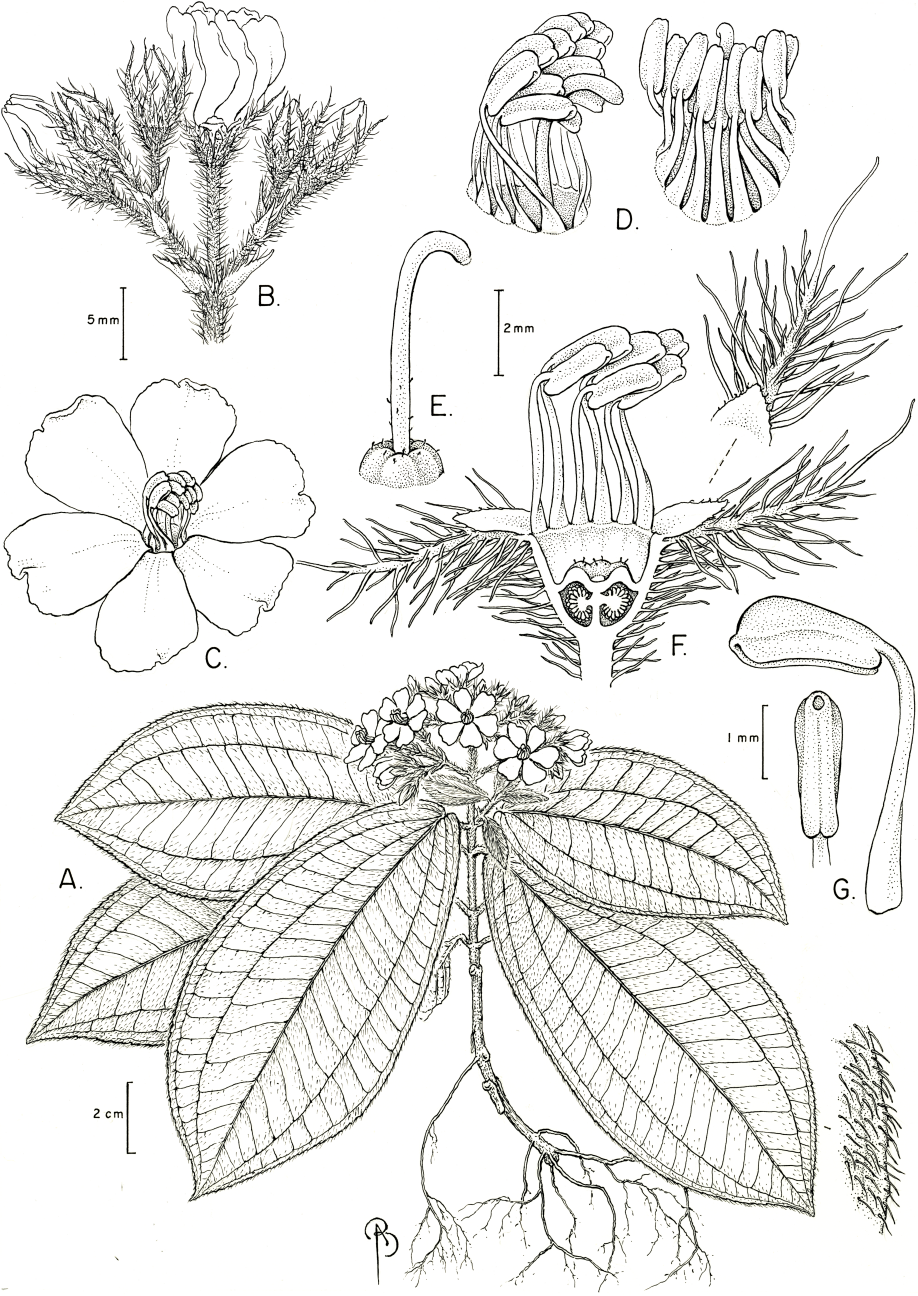
*Physeterostemon aonae*. (A) Habit. (B) Inflorescence with buds and flowers at anthesis. (C) Flower, frontal view. (D) Details of stamen disposition at anthesis, lateral and dorsal view. (E) Ovary apex and style. (F) Flower, longitudinal section with petals removed. (G) Stamens in ventral and lateral view (all drawn from Amorim 8885, NY, alcohol-preserved flowers and photos of live plants).

**Type**. Brazil, Bahia: Mun. Wenceslau Guimarães, Povoado de Nova Esperança, Assentamento Patioba, 13°34′44.5^′′^S, 39°41′44.0^′′^W, 520 m, 01 Dec 2014 (fl), *A. M. Amorim, J. L. Paixão* & *L. C. Marinho 8885* (holotype: CEPEC; isotypes: NY, RB, UPCB).

**Diagnosis**. A species of *Physeterostemon* similar to *P. jardimii* R. Goldenb. & Amorim but with leaf base rounded to subcordate, inflorescences paniculate/corymbose, hypanthia strigose but not glandulose-setose, calyx with sepals 1.9–2.1 mm long and dorsal teeth 4.1–4.3 mm long, petals 7.7–8.4 mm long.

**Description**. Rhizomatous subshrub 12–20 cm high. Young stems and petioles densely covered with trichomes 0.7–2 mm long, appressed, unbranched, with broadened bases; inflorescences, hypanthia and calyx with similar, but erect or curved trichomes 2–3 mm long. Young stems slightly terete. Leaves opposite, isomorphic; petioles 0.5–2.3 cm long; blades (5-)8–21 × (2-)4–8 cm, membranaceous, oblong or oblanceolate to elliptic-lanceolate, base rounded to subcordate, frequently asymmetric, apex broadly acuminate or acute, margin crenulate, denticulate to serrate; venation acrodromous, suprabasal, lacking domatia, with one or two pairs of secondaries diverging 3–12 mm above the base and with an additional pair of faint submarginal veins, tertiary veins percurrent, adaxial surface moderately covered with trichomes 1–1.5 mm, appressed, the main veins impressed, tertiaries slightly impressed, quaternaries barely visible, reticulation not visible, abaxial surface actually glabrous, but with trichomes on main and secondary nerves, the trichomes similar to the ones on the branches, and also smaller and erect trichomes on the tertiary veins (ca. 1 mm long) and quaternary veins and reticulation (0.3–06 mm long), main, secondary and tertiary veins prominent, quaternaries and reticulation plane. Inflorescence 4.3–5.4 × 4.2–6.5 cm, terminal, erect, usually a single corymbose panicle with 2–4 pairs of paraclades, 20–30 flowers, these congested at the apices of the branches, the axes vinose; bracts 1.5–3.2 × 1.1–1.7 mm, deltoid to lanceolate, margins ciliate, adaxial surface glabrous, abaxial glabrous but with the main vein slightly elevated and with a few trichomes on it; bracteoles 1.8–2 × 0.6–0.8 × 1–2 mm, similar to the bracts. Flowers (5-)6-merous, the pedicels 2–4.8 mm long. Hypanthium ca. 3 × 3.5 mm, green but covered with reddish to vinose trichomes, terete to oblong-urceolate, internal surface and torus glabrous. Calyx green but covered with reddish to vinose trichomes, opening regularly; tube ca. 0.8 mm long; sepals 1.9–2.1 mm long, deltoid to rounded, margins shortly ciliolate (cilia ca. 0.6 mm long); dorsal teeth 4.1–4.3 mm long, linear. Petals 7.7–8.4 × 4.6–5.4 mm, white (but light-pink in the buds), obovate, apex truncate, margins entire, abaxial surface slightly papillose. Stamens (10-) 12, isomorphic, all grouped in a cluster at one side of the flower at anthesis; filaments 3.1–3.5 mm long; connective unappendaged, dorsally thickened; thecae 2–2.1 mm long, with a single ventral pore 01–02 mm diameter. Ovary 1.4–2 mm long, 1/2–2/3 inferior, 4-locular, apex sparsely covered with short glandular trichomes ca. 0.1 mm long; style 5–5.3 mm long, pale-yellow, straight but with a curved apex, sometimes with very scattered glandular trichomes similar to the ones on the ovary apex,; stigma punctiform. Capsules 2.4–3.2 × 3.6–4 mm, tearing longitudinally, with the pericarp adnate to the hypanthium, and both persistent; seeds not seen.

**Distribution and Conservation Status**. *Physeterostemon aonae* is known from only one population near the “Estação Ecológica Estadual Wenceslau Guimarães” in southern Bahia. This single population is located outside the reserve in a slope side near a cocoa plantation. Searches for this species in similar habitats within the reserve and other nearby forest patches failed to find another population. Given the restricted size of the known habitat, we recommend that *P. aonae* is given a status of “critically endangered” (IUCN, 2014).

**Paratypes**. Brazil, Bahia: Mun. Wenceslau Guimarães, Estação Ecológica, Assentamento Patioba, 11 Mar 2013 (fr), *L.Y.S. Aona et al. 2388* (CEPEC, HURB, K, RB, UPCB); Mun. Wenceslau Guimarães, Nova Esperança, Patioba, 13°34′44.5^′′^S, 39°41′44.0^′′^W, 520 m, 20 Feb 2014 (fr), *R. Goldenberg et al. 1740* (CEPEC, NY, UPCB); Mun. Wenceslau Guimarães, Nova Esperança, Patioba, 13°34′44.5^′′^S, 39°41′44.0^′′^W, 520 m, 11 Oct 2014 (st), *R. Goldenberg & F.A. Michelangeli 2041* (HURB, UPCB); Mun. Wenceslau Guimarães, Nova Esperança, Patioba, 13°34′44.5^′′^S, 39°41′44.0″W, 520 m, 01 Dec 2014 (fl), *A. M. Amorim et al. 8886* (CEPEC); Mun. Wenceslau Guimarães, Nova Esperança, Patioba, 13°34′43″S, 39°41′36″W, 444 m, 19 Dec 2015, *L. Bacci et al. 287* (CEPEC, HUESC, NY, RB, UEC, UPCB).

**Remarks**. *Physeterostemon aonae* is similar to *P. jardimii*, but can be distinguished by the blade rounded to subcordate at the base (*vs.* acute to narrowly rounded in *P. jardimii*), hypanthium strigose but not glandulose-setose (*vs.* strigose and glandular-setose), sepals 1.9–2.1 mm long (*vs*. 1.2–1.3 mm long), dorsal teeth 4.1–4.3 mm long (*vs.* 2.9–4.1 mm long) and petals 7.7–8.4 mm long (*vs.* ca. 7 mm long).

## Additional Information and Declarations

### Field Study Permissions

The following information was supplied relating to field study approvals (i.e., approving body and any reference numbers): INEMA/BA (2014-008132/TEC/PESQ-0010 and 2015-010036/TEC/PESQ-0015).

### Data Availability

The following information was supplied regarding data availability: The research in this article did not generate any raw data.

### New Species Registration

The following information was supplied regarding the registration of a newly described species: *Huberia sessilifolia* R. Goldenb. & Michelangeli, *H. sessilifolia* 77153600-1; Meriania *inflata* Michelangeli & R. Goldenb., *M. inflata* 77153601-1; *Physeterostemon aonae* Amorim, Michelangeli & R. Goldenb., *P. aonae* 77153602-1.
